# Ecological correlates of sociality in *Pemphigus *aphids, with a partial phylogeny of the genus

**DOI:** 10.1186/1471-2148-7-185

**Published:** 2007-10-03

**Authors:** Nathan Pike, John A Whitfield, William A Foster

**Affiliations:** 1 Department of Zoology, University of Oxford, South Parks Road, Oxford OX13PS, UK; 2 Department of Zoology, University of Cambridge, Downing Street, Cambridge CB23EJ, UK

## Abstract

**Background:**

Because the systems of social organisation in the various species of *Pemphigus *aphids span the continuum from asociality through to advanced sociality (typified by the possession of morphologically specialised soldiers), the genus is an ideal model clade in which to study the influence of ecology on the origins of eusociality. We made detailed study of the ecology of three gall-dwelling species that show clear differences in their levels of social behaviour. To elucidate evolutionary relationships and to attempt to estimate the number of origins of sociality, we also created a phylogeny based on sequences spanning the mitochondrial genes Cytochrome Oxidase I and II for nine species of *Pemphigus*.

**Results:**

*P. spyrothecae*, a highly social species with aggressive morphologically-specialised soldiers, has the longest galling phase, unsynchronised development of a large number of individuals in a densely-populated gall, and an extended period over which alates emerge. *P. populi*, a species with no soldiers, has the shortest galling phase, synchronised development of a small number of individuals in a sparsely-populated gall, and an extremely brief emergence period. The ecology of *P. bursarius*, which has behavioural soldiers that are not morphologically specialised, is intermediate between these two extremes. The galls of *P. spyrothecae *and *P. bursarius *form small openings during the course of the season and predation-related mortality is relatively high in these two species. Conversely, predation does not occur during the galling phase of *P. populi*, which has no soldiers but makes an entirely-sealed gall.

The phylogeny allowed us to infer one likely point of origin of basic social defence and two independent origins of enhanced defence. Based on current knowledge of behaviour, the phylogeny also suggests that the defence trait may have been lost at least once.

**Conclusion:**

The life-history strategy of *P. spyrothecae *appears to be geared towards defending the colony against the constant threat of predation that faces the inhabitants of a long-lived, open gall. The life-history strategy of *P. populi*, on the other hand, is to avoid predation in the closed gall fortress and flee for the secondary host at the earliest opportunity. The life-history strategy of *P. bursarius *appears to represent a compromise between these strategies.

## Background

The transition from solitary to social living is one of the key events in evolution: the rarity of this transition has provided a classic problem for evolutionary biologists and the unparalleled success of social animals is of central interest to all ecologists. To investigate the factors underlying this transition, it must be studied in a phylogenetic context and we therefore require clades of extant species that have several independent origins and different degrees of sociality. Such clades are exceptionally rare. For many groups, the number of independent origins of sociality is very small, these origins may be buried deep in the past, and the phylogeny may not be known. Other groups, for example bees and thrips, are more promising. The social aphids provide particularly fruitful material because there have been many independent origins of sociality and there are clades of known phylogeny containing species covering the spectrum from solitary to fully social organisation. There are estimated to be at least 17 independent origins of sociality in aphids (T. Fukatsu, pers. comm. 2006) even though only 1% of aphid species is social [1,2]. This dwarfs the equivalent estimates for other social taxa (e.g. 1 origin in the ants [3], 1–4 origins in the termites [4], fewer than 11 times in the wasps and bees [5]). In addition, aphids have a genetic system – clonality – that is virtually unique among social insects. This reduces the complexity caused by genetic conflict that is present in most social systems and allows us to concentrate on the role of ecological factors, which have probably driven the evolution of all animal societies [6].

The vast majority of aphid species simultaneously produce more than one morph, and the different morphs routinely have different direct reproductive fitnesses. For example, wingless morphs may produce more offspring than winged morphs [7]. It is certainly true that such reproductive sacrifice may constitute an altruistic act which enables the altruist to contribute indirectly to the fitness of its clonemates though behaviours such as migration. However, in line with the approach most commonly taken, we exclude such altruism from our definition of true aphid sociality, which we consider to have occurred only when a species has acquired soldiers, morphs that actively defend against predators. It is undoubtedly relevant that these soldiers are often the agents of socially advanced behaviours other than defence, such as nest cleaning [8,9], nest repair [10,11] and invasion and exploitation of alien colonies [12].

Every species of social aphid forms and inhabits a plant gall at some point in its life cycle [13]. The galls provide a rich and indispensable resource to the aphids which are therefore classic examples of "fortress defenders" [14]. The gall is especially important for serving as a physical barrier to predators and to alien aphid invaders that seek to exploit the altruism of the home clone, disrupting the alignment of genetic interests among colony mates.

This study concerns the aphid genus *Pemphigus *which contains species that exhibit almost the entire spectrum of sociality from the total absence of defenders to the presence of aggressive, morphologically specialised soldiers [15]. *Pemphigus *soldiers generally occur in the first instar of the virginoparous morph on the primary host but defensive behaviour also continues to a lesser degree in later virginoparous instars [15]. It is clear that the gall is particularly crucial to the social biology of *Pemphigus *aphids because soldier morphs have been found only at the galling stage [16]. We produce a partial phylogeny that includes nine of the sixty-five species of *Pemphigus *that have been described [2]. The species of the genus are spread across the Northern Hemisphere and many have cosmopolitan distributions [17]. *Populus *(poplar) is the primary host on which the galls are formed [18] and host alternation is the norm, but not the rule: three of the 17 species that have been sufficiently studied spend their entire life cycle on poplar. Because the genus is entirely without secondary-host soldiers, we can exclude the possibility that a second, dissimilar environment influences social evolution and we can thus focus solely on the galling phase. The *Pemphigus *species that have been scrutinised for the presence of soldiers are presented in Table 1. In this study, we have examined in detail the natural history and ecology of three species: *Pemphigus populi*, which does not have soldiers; *Pemphigus bursarius*, which invests moderately in gall defence using monomorphic nymphs that behave as soldiers; and *Pemphigus spyrothecae*, which invests extensively in morphologically specialised soldiers.

**Table 1:** The species of *Pemphigus *in which soldiers have been sought

**Species**	**Distribution**	**Host-alternating?**	**Soldiers present?**	**Reference**
***P. bursarius ***(Linnaeus)	Worldwide	Yes	**Yes**	[15]
*P. dorocola *Matsumura	Japan, Korea, Siberia	Yes	**Yes**	[23, 46]
*P. gairi *Stroyan	UK	Yes	**Yes**	[15]
*P. monophagus *Maxson	Western North America	No	**Yes**	[31]
*P. obesinymphae *Aoki & Moran	USA	Yes	**Yes**	[47, 48]
*P. phenax *Börner & Blunck	Europe	Yes	**Yes**	[15]
***P. populi ***Courchet	Europe, Asia	Yes	**No**	[15]
*P. protospirae *Lichtenstein	Europe, central Asia	Yes	**Yes**	[15]
***P. spyrothecae ***Passerini	Europe to westernSiberia	No	**Yes**	[22, 24]

The three species that were subject to detailed ecological study are shown in bold typeface. (After Rhoden and Foster [15])

Within the genus *Pemphigus *there is known to be considerable variation in factors such as time spent on the primary host, the number of aphids within a gall, and the number of emigrants produced during the galling phase [18]. By examining these and other ecological, demographic and phenological differences (with special reference to measures of mortality, caste composition, and timing of gall opening), our aim is to elucidate the key selective factors that have influenced the evolution of sociality in the galling aphids and other fortress-defending organisms.

## Results

### Ecology of *P. populi*

The average gall volume at the end of *P. populi*'s galling phase was 1099 ± 458 mm^3^. Larger galls tended to contain greater aphid populations, increasing by approximately 1 aphid for every 70 mm^3 ^of gall volume (*R*^2 ^= 0.31, *F*_1,19 _= 8.62, *P *= 0.008). Correspondingly, larger galls also tended to allow the emergence of increased numbers of alate fundatrigeniae (with the number of these emigrants estimated to increase by approximately 1 for every 50 mm^3 ^of gall volume, *R*^2 ^= 0.21, *F*_1,25 _= 6.972, *P *= 0.014).

The index of developmental synchronisation in the gall of *P. populi *was calculated for all the destructive samples because the full range of instars was never in the gall at the same time. There was no significant difference between the resulting indices for each sample (*F*_10,40 _= 0.69, *P *= 0.72). The index calculated from the pooled samples indicated a high level of developmental synchronisation: on average, 0.92 ± 0.02 of the individuals in a gall was belonged within the same single instar.

The average number of fundatrigeniae in destructively-sampled galls was 42.9 ± 20.3. (The fundatrigeniae were the only morph in the gall other than the foundress.) This value accorded well with the average number of alate fundatrigeniae that left the monitored gall and were captured in bags: 41.0 ± 21.2. The density of aphids in galls of *P. populi *at the end of the season is therefore about 0.03 aphids mm^-3^.

**Figure 1:**
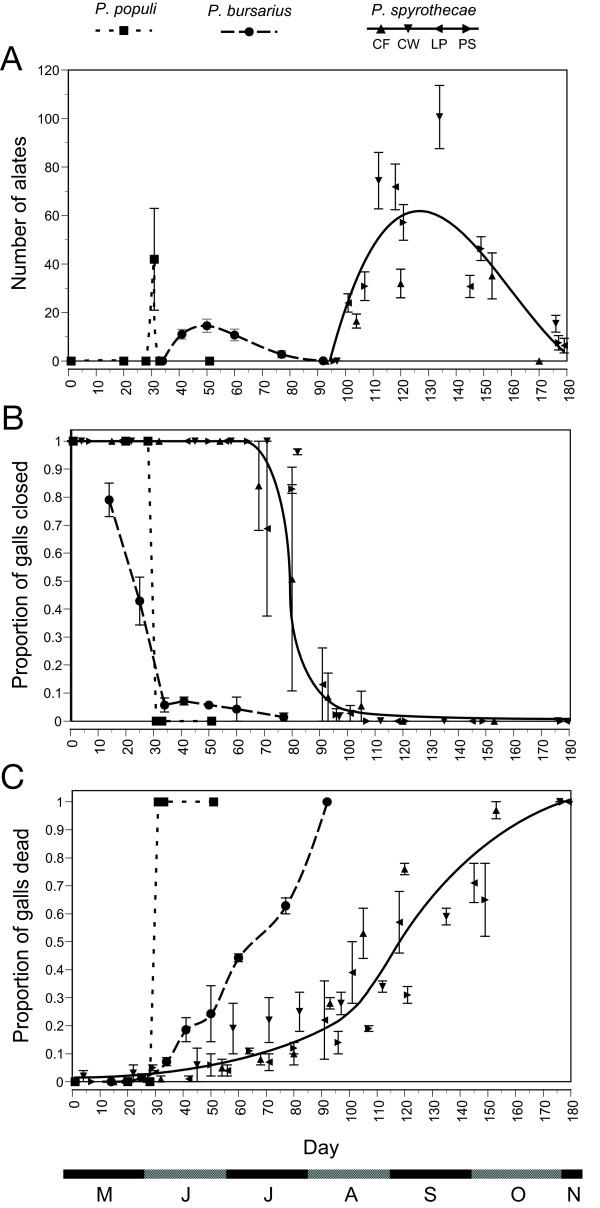
**Comparison of the phenological characteristics of the three species *P. populi*, *P. bursarius *and *P spyrothecae***. (A) the emergence patterns, (B) the patterns of gall opening, and (C) the patterns of gall mortality (death of all the individuals within the gall). Splines are fitted to the data of *P. populi *and *P. bursarius *and regression curves are plotted for the combined data for *P. spyrothecae*. The data from the different field sites from which *P. spyrothecae *was sampled (CW: Cement Works, LP: Leys Playing Field, PS: Pembroke Sportsground, CF: Coton Footpath) are identified by the unique triangular symbols indicated in the legend.

The opening of the galls of *P. populi *is highly unusual. Wide fissures open in the gall starting at its apex and often extend so far that the gall's inner surface is turned entirely outward. A closed and an open gall are depicted in Table 2. Alate fundatrigeniae (which comprised virtually the entire aphid population) emerged immediately that the galls opened. This emergence was synchronised: the fundatrigeniae of all the bagged galls emerged on the same day, June 10 (see Figure 1A).

**Table 2:** Differences among *P. populi*, *P. bursarius *and *P. spyrothecae *in key defence-related components of their life history strategies

**Trait**	***P. populi***	***P. bursarius***	***P. spyrothecae***
**Gall morphology**			
	mean volume = 1099 ± 458 mm^3^	mean volume = 521.0 ± 45.7 mm^3^	mean volume = 636.7 ± 13.4 mm^3^
**Aphid density**	Low: 0.03 aphidsmm^-3^	Moderate: 0.11 aphidsmm^-3^	High: 0.35 aphids mm^-3^
**Galling period**	Short: ~2 months	Moderate: ~3 months	Long: 5–6 months
**Demographic synchrony** (Highest proportion represented by a single morph)	Most synchronous: 0.92 ± 0.02	Moderately synchronous: 0.67 ± 0.02	Least synchronous: 0.43 ± 0.01
**Period of alate departure**	Short: 1–2 days	Moderate: 30–40 days	Long: ~60 days
**Reproductive success** (number of alates/emigrants)	Lowest: 41.03 ± 21.18	Intermediate: 59.4 ± 8.8	Highest: 128.6 ± 8.5
**First instar**	Monomorphic with no soldiers	Monomorphic with moderately aggressive behavioural soldiers	Dimorphic with highly aggressive soldier morph

**Overall strategy**	Hide and run	Stay a little, fight a little, but then run	Stay and fight

Immediately following emigration from the gall of the fundatrigeniae (June 11), 42.0% of galls contained predators, 47.3% contained inquiline aphids, and 22.7% contained aphidopathogenic fungus. 87.3 % of the monitored galls (131 out of 150) were thus affected by phenomena that would have been fatal to aphids had they continued to inhabit them. This extremely high rate of gall mortality is shown in Figure 1C.

### Ecology of *P. bursarius*

The average volume of *P. bursarius *galls at the time of peak population (June 20) was 521.0 ± 45.7 mm^3^. Galls with larger volumes again tended to contain larger numbers of aphids, with the aphid population increasing by approximately 1 for every 9 mm^3 ^increase in volume (*R*^2 ^= 0.45, *F*_1,17 _= 13.78, *P *< 0.001). The trend for a greater number of alate fundatrigeniae to emerge from larger galls was not significant (*F*_1,28 _= 3.39, *P *= 0.08).

**Figure 2:**
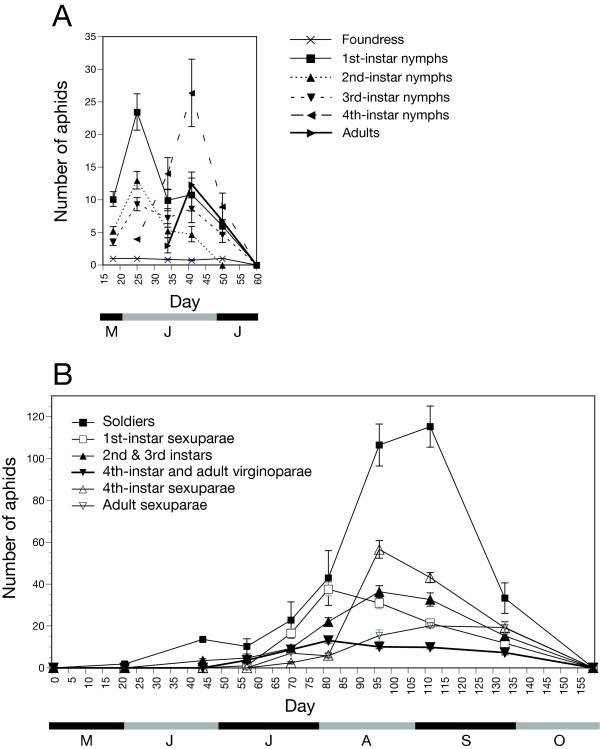
**Demographic composition of destructively-sampled galls**. (A) *P. bursarius*: breakdown of aphid instars (mean number per gall ± SE) over the duration of the galling season; (B) *P. spyrothecae*: breakdown of aphid morphs/instars (mean number per gall ± SE) at the Cement Works field site over the duration of the galling season. Note that the y-axes are on different scales.

As the average number of aphids in galls at peak population was 57.9 ± 10.9, the density of aphids in the galls of *P. bursarius *was approximately 0.11 aphids mm^-3^. The fourth-instar predominated at the time of peak population. The index value for synchronisation of aphid development was 0.67 ± 0.02. The galls were empty by July9, approximately 2.5 months after they had been formed (see Figure 2A).

The galls of *P. bursarius *were open for most of the galling phase. Galls were closed for an initial 2–4 weeks but virtually all had opened by mid-June (Figure 1B). The approximately circular openings measured 1–1.5 mm in diameter. Emergence of alate fundatrigeniae began in mid-June and had concluded by the end of July (Figure 1A). Peak emergence was in early July. The mean number of fundatrigeniae emerging from each gall was 59.4 ± 8.8 for the entire season.

The rate of gall mortality was approximately constant over the entire galling phase (Figure 1C). The increase in mortality in response to opening of the galls was not especially pronounced because opening occurred very near to the beginning of the galling phase when failures (unrelated to predation) of the small founding populations were at their highest.

### Ecology of *P. spyrothecae*

The total number of resident aphids within a gall correlated positively with gall volume in all cases. For example, at Pembroke Sportsground for the galls collected over the sampling period encompassing 14 August, 26 August and 8 September, 1999, the aphid population tended to be larger by one for every 3.5 mm^3 ^increase in gall volume (*R*^2 ^= 0.24, *F*_1,131 _= 40.36, *P *< 0.001). A positive correlation with volume was also true for the number of sexuparae emerging from the galls, which tended to increase by one for every 5 mm^3 ^increase in gall volume (pooled result for all four sites: *R*^2 ^= 0.33, *F*_1,74 _= 35.96, *P *< 0.001).

The aphid population peak occurred in mid-August. Pooling the two peak-population sampling events for every site, the average number of aphids per gall is 222.5 ± 6.4. The average gall volume at this time is 636.7 ± 13.4 mm^3^. The peak density of aphids inside the gall is therefore around 0.35 aphids mm^-3^. Soldiers and mature sexuparae were particularly numerous at the time of population peak and the peak in population co-incided with the opening of the galls. Fluctuations in the numbers of both virginoparous and sexuparous morphs over the entire field season are presented for each site in Figure 2B.

Aphid population size was influenced by the same factors that affected gall volume. Analysis of variance using the statistical model

Number of aphids = day + day × height + day × site(tree) + day × height × site(tree) + *ε*

showed that the first order interaction of *day *with *site *was highly significant (*F*_3,1504 _= 27.76, *P *< 0.001), just as population differences among trees remained detectable after having accounted for time-dependent and site-dependent differences (*F*_4,1504 _= 13.92, *P *< 0.001). Again, the height from which the galls were collected was not found to have a significant effect (*P *> 0.1 in both cases).

There was no relationship between the proportion of soldiers in a mature colony and colony population size at any site. Similarly, there was no relationship between the proportion of soldiers in a mature colony and gall size.

For *P. spyrothecae*, the index of developmental synchronisation was calculated at the time when all morphs were well-represented in the gall (August). First-instar soldiers predominated, making up 0.43 ± 0.01 of the gall. Death of a colony's foundress usually occurred 2–4 months after the gall's initiation. In mid-August (Day 90), approximately half of the galls contained a living foundress but all foundresses were dead by mid September (Day120).

Adult sexuparae began to emerge from the galls immediately that the openings were formed (see Figures 1A and 1B). The elliptical openings ranged from 1–2 mm in length (measured along the axis of the gall spiral) and 1–1.5 mm in width. Emergence continued for a period of almost three months. Peak emergence occurred in the month of September at the middle of the emergence period. The average number of sexuparae that emerged from each gall over the course of the season is 128 ± 8.5. Gall mortality (i.e. when all the aphids died) increased markedly following the opening of the galls, with the rate of gall mortality at least doubling in all cases (Figure 1C).

### Phylogeny

**Figure 3:**
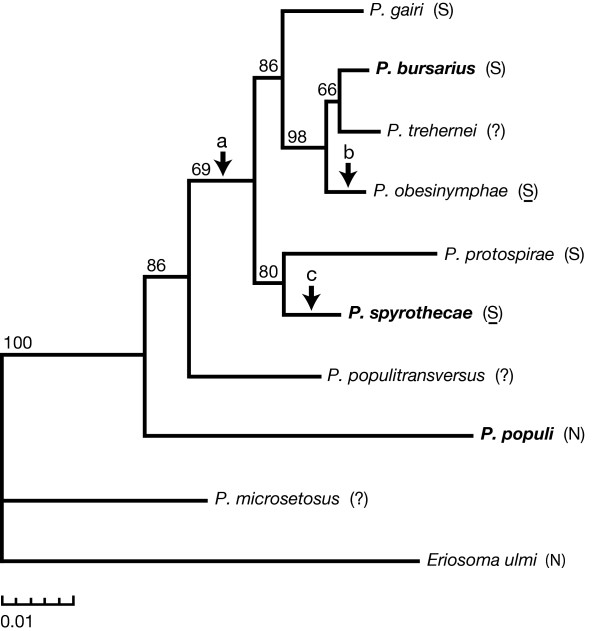
**Phylogram of nine species of *Pemphigus***. The three species that were subject to detailed ecological study are shown in bold typeface. *Eriosoma ulmi *is the outgroup. A parenthetical "S" indicates that the species possesses soldiers and, where underlined, highly aggressive soldiers with specialised defensive morphology. The absence of soldiers is denoted by a parenthetical "N". Arrow (A) indicates the likely origin of soldiers, arrow (B) indicates the evolution of highly aggressive soldiers, and arrow (C) indicates the evolution of highly aggressive soldiers along with loss of host-alternation and the gain of dimorphic nymphs. The numbers on the branches give the number of times out of 100 that a bootstrap replicate recovered the branch, and the scale indicates units of 0.01 expected substitutions per nucleotide position.

All four phylogenetic techniques produced the same topology which is given in Figure 3. Maximum likelihood bootstrapping analysis gives this tree topology strong support. Bayesian analysis estimated the posterior probability of this topology to be 0.82. The tree with the next greatest posterior probability of 0.12 differed in just one respect: the positions of *P. populitransversus *and *P. populi *were exchanged.

Bayesian inference was also used to test support for monophyly of species known to possess soldiers. In the unconstrained analysis just described, if we assume that *P. trehernei *and *P. populitransversus *both possess soldiers, the cumulative posterior probability of a phylogeny containing a clade which is monophyletic for presence of soldiers is 0.95. On the other hand, the same analysis estimates that there is zero probability of a tree with soldier monophyly if we assume that neither *P. trehernei *nor *P. populitransversus *possesses soldiers. A second analysis which constrained *P. gairi*, *P. bursarius*, *P. obesinymphae*, *P. protospriae*, and *P. spyrothecae *to be monophyletic was also conducted. The difference in the harmonic means of the likelihood values from each of the two analyses was used to estimate the Bayes factor of 27.88. This value provides strong evidence that the model with the constraint of a five-species monophyletic soldier clade should not be accepted over the unconstrained model [19]. However, the difference resulting from comparison of the unconstrained model to a constrained model which adds *P. trehernei *as the sixth species of the monophyletic clade is less marked, as indicated by the considerably weaker Bayes factor of 12.48.

## Discussion

### Life-history and ecological traits

Detailed ecological investigation of the three study species provided a clear demonstration of which factors are likely to have had the predominant influence in producing divergent defensive strategies through evolutionary optimisation of the aphids' life histories (Table 2).

#### Duration of the galling phase

The duration of the galling phase was approximately two months in *P. populi*, approximately three months in *P. bursarius*, and between five and six months in *P. spyrothecae*. *P. populi *is thus exposed to the selective environment of the primary host for the shortest time, while *P. spyrothecae *is exposed for the longest.

#### Aphid density

The lowest aphid density occurred in the galls of *P. populi *and was up to four times greater in *P. bursarius *galls and up to twelve times greater in *P. spyrothecae *galls. These density figures also reflect interspecific differences in the size of aphid populations supported in a gall. Whereas the *P. bursarius *peak populations (57.9 ± 10.9) were only slightly bigger than those of *P. populi *(42.9 ± 20.3), the peak populations of *P. spyrothecae *were more than four times the size (222.5 ± 6.4). Population size and density are likely to relate to factors such as the duration of the galling phase and the robustness of a colony following negative influences such as predation. Population size and density are also likely to have an integral influence on gall hygiene. It was noted that detritus such as moulted cuticle and waste such as wax-covered honeydew accumulated in the bottom of galls of *P. populi*. This was not the case for galls of *P. bursarius *and *P. spyrothecae*, the inhabitants of which push detritus and waste out of the holes in their galls [8,9].

#### Gall opening

The opening of the gall saw a rise in aphid mortality in all three of the species. This opening releases a colony's winged migrants, but it also compromises the integrity of the gall as a physical boundary to predation [11] and, in *P. spyrothecae*, is the reference point for the specialised spatial distributions of the various gall morphs [20]. The potential risk of predation seems especially high for *P. populi*, the species without soldiers. Although alate fundatrigeniae of this species departed from the gall immediately that it opened, almost 90% of the galls suffered from potentially disastrous incursions by predators, inquilines and pathogens on the day of opening. Whereas the opening of galls of *P. populi *takes the form of extensive rupturing of the gall, opening in *P. spyrothecae *and *P. bursarius *is a more subtle process which involves the formation of a small hole (<2 mm in diameter) in the otherwise sealed gall. The presence and size of these openings, is known to be a crucial factor which the aphids closely regulate and, if necessary, repair to minimise predation [11].

#### Alate emergence

Each of the three species had a unique time for emergence, and there was little overlap among the emergence periods. The emergence of *P. populi *occurs first, in early June, and is followed by *P. bursarius *in June-July and finally by *P. spyrothecae *in August-October. There is also a striking difference between the durations of the emergence periods. *P. populi *emerge in a single pulse, with all the winged fundatrigeniae leaving their galls in virtually one day. The fundatrigeniae of *P. bursarius *emerge over a period of up to two months, while emergence of the sexuparae of *P. spyrothecae *can continue for more than three months (Figure 1).

#### Synchronisation of development

The rapid emergence of *P. populi *in particular, but also of *P. bursarius*, is facilitated by a high degree of developmental synchronisation. More than 90% of the aphids within a gall of *P. populi *tended to be of the same instar and up to 60% were of the same instar in galls of *P. bursarius*. Being from host-alternating species, these instars were soon destined to migrate from the gall. In contrast, first-instar soldiers (none of which are destined to emerge) comprised the greatest single morph in galls of *P. spyrothecae*. Despite being the most prevalent morph, the concurrent presence of a number of other morphs meant that these soldiers accounted only for approximately 40% of aphids.

### Phylogenetic inferences

The three species that were subject to detailed ecological study are spread across the partial phylogenetic tree presented in Figure 3. The likely origin for the evolution of defensive behaviour is at the branch (point A) which gives rise to the six uppermost species of the tree. Behavioural defence is known for five of these six species, with *P. trehernei *representing the single exception. The loss of defensive behaviour on the *P. trehernei *branch must remain putative until thorough investigation is made but, if confirmed, the loss is likely to be associated with a life-history shift away from the primary host: the species has not been found to form galls in natural conditions [21]. Although the partial phylogeny suggests just one origin and one loss of the basic defence trait, it also suggests that pronounced defence through highly aggressive soldiers has evolved twice, once in the case of *P. obesinymphae *and once in the case of *P. spyrothecae *(points B and C on Figure 3). In the latter species, this increased defence is likely to have been supported by the evolution of dimorphism and the loss of all secondary hosts. The phylogeny certainly supports the hypothesis that *P. spyrothecae *evolved from a host-alternating ancestor [22].

Although detailed consideration of the evolution of gall forms (beyond their defensive implications) is outside of the scope of the current work, we note that the gall morphologies described by Blackman and Eastop [17] correspond well with the groups borne out in our molecular phylogeny. There is evidence of phylogenetic conservation of gall morphology in the facts that *P. gairi *has an elongate midrib gall, *P. bursarius*, *P. obesinymphae *and *P. populitransversus *have round petiole galls, while spiral petiole galls occur only in the *P. protospirae*/*P. spyrothecae *subgroup which is therefore probably monophyletic. The apparent phylogenetic conservation of gall morphology may also indicate the likely position of several species that are currently absent from our tree. For example, species such as *P. matsamurai *have galls that, like those of the study species *P. microsetosus *and *P. populi*, form on the leaf and dehisce in a secondary position to the original opening. On the other hand, with the possible exception of *P. trehernei *(which apparently has a unique biology and forms galls only rarely), our phylogeny does not include the various species of *Pemphigus *that form twig galls. These species are likely to belong to a separate subgroup, the position of which is yet to be elucidated. Intriguingly, because this subgroup would include *P. dorocola*, a species known to have soldiers [23], a second independent origin of defensive behaviour would be required.

The species examined in our phylogeny represent merely a small proportion of the large number of *Pemphigus *species and those of other related genera. Monoecy and defence by soldiers is certainly known to occur in a number of species that were not included in the current research. Incorporating these species into future studies will facilitate the expansion and, possibly, the amendment of the evolutionary relationships that we have inferred. Broader phylogenetic knowledge combined with improved behavioural and ecological knowledge will undoubtedly provide answers to some of the most interesting questions regarding the relationship between the selective environment and trends in social evolution.

## Conclusion

### Life-history strategies

#### Hide and run

*P. populi *has a short galling phase. The short time in the gall means that the time exposed to the predators of the primary host is reduced. Maintaining a completely sealed gall further isolates the colony from external influences. The build-up of waste is not a problem because colonies are relatively small and sparsely populated. Because the closed gall provides an extremely effective barrier to predation, soldiers are not required. The only time that the colonies are susceptible to predation is during the emergence period. This period is kept extremely brief so that predation is minimised. The pulse of emergence is facilitated by synchronising the development of the instars so that all are mature alate fundatrigeniae at the time of gall opening.

#### Stay and fight

*P. spyrothecae *has a long galling phase and a long emergence period. These extended periods can be sustained because the species possesses morphologically specialised, aggressive soldiers that are able to defend their colonies against predation. The long time in the gall under the protection of soldiers means that it is possible to increase a colony's reproductive success by having multiple generations and staggered development during the galling phase. The colony size is larger than those of the other species and aphids of *P. spyrothecae *live at a high density within their galls. The problem of the accumulation of waste at such high densities is controlled by having an opening to the gall by which the waste can be removed. The opening of the gall is relatively small and can be effectively guarded by the soldiers. A sustainable trade-off between preventing predators from entering and allowing migrants and waste to leave is thus possible.

#### A mixed strategy

*P. bursarius *has a galling phase and emergence period of moderate length. These periods of exposure can be borne by having moderately aggressive behavioural, but not morphological, soldiers. Galls have a small ostiole for most of the galling period. As is the case for *P. spyrothecae*, the presence of these ostioles can be supported because a balance can be found between ejecting waste and detritus from the gall and repelling predators with soldiers. The moderately high density of aphids makes this waste removal advantageous. Prolongation of the emergence period is prevented because the development of the immature aphids, which are all offspring of the foundress, is largely synchronised.

### Key correlates of sociality

A variety of factors likely to be significant in the evolution of aphid soldiers have been identified. Increased defence investment is correlated with increasing aphid density, longer galling periods, longer periods of alate departure, small gall openings, and decreasing developmental synchrony. It is certain that predation pressure, an understudied factor, will have the central role in the evolution of defence. This assertion has been borne out, albeit in somewhat general terms, by the empirical (e.g. [11,24,25]) and theoretical studies that have dealt with the issue to date (e.g. [26-30]).

Another pivotal factor influencing the degree of defensive specialisation is whether loss of host alternation or other factors have influenced each species' innate predisposition for producing dimorphic offspring. Because dimorphism can facilitate the evolution of specialised defenders, it may well also result in increased fitness. Particularly in the genus *Pemphigus*, the loss of host alternation is likely to be a key determinate of this predisposition to dimorphism [22]. *P. spyrothecae *is the only study species that is not host-alternating and the loss of host alternation is likely to have increased the length of its galling phase. Certainly, other species that are restricted to the primary host, such as *P. monophagus *[31], also spend a relatively long period in the gall and have dimorphic first instars.

We hope that, by way of example, the current study has succeeded in exposing the excellent prospects that the genus *Pemphigus *holds as a model clade that can advance our understanding of the non-genetic influences upon the continuum that is sociality.

## Methods

### Study species

#### Typical *Pemphigus *life cycle

The majority of *Pemphigus *species have host-alternating life cycles in which periods spent on the woody primary host (poplar trees; *Populus nigra *in all three species described below) are succeeded by periods spent on herbaceous secondary host plants [17]. In spring, the founding female hatches from an egg which overwintered on the bark of the primary host and this foundress initiates a gall on the poplar leaf which is species-specific in its architecture. The foundress parthenogenetically produces the gall generation of tens to hundreds of individuals which, when mature, are specialised winged morphs (called fundatrigeniae/primary-host virginoparae) which migrate to the secondary hosts. Upon reaching the secondary host, the fundatrigeniae give rise (asexually) to wingless morphs (called virginoparae) which may dwell on the roots for a number of asexual generations before producing other winged migrant morphs (called sexuparae) which return to the primary host where they give rise to both male and female offspring. After mating, each female produces a single overwintering egg.

#### *Pemphigus populi *Courchet, 1879

The life cycle of *P. populi *adheres closely to the typical life cycle [32]. The monomorphic nymphs of *P. populi *exhibit no defensive behaviour [15], just as no other social trait has been found in this species.

#### *Pemphigus bursarius *(Linnaeus, 1758)

This species also has a life cycle that is broadly typical [33,34]. However, the wingless secondary host females may occasionally produce a unique winged morph that can fly from one secondary host plant to another where it may found a new colony directly. The usual survival requirement of returning to primary host can thus be bypassed entirely [35].

Although the fundatrigenious nymphs of *P. bursarius *are monomorphic and without obvious morphologically specialised defenders, the first-instar individuals exhibit defensive behaviour. They respond aggressively to the cue of aphid haemolymph and actively attack potentially predacious syrphid larvae with their claws and stylets [15,16].

#### *Pemphigus spyrothecae *Passerini, 1856

The species has an atypical life cycle which is spent entirely on the primary host. Inside the gall, the foundress gives birth to a generation of aphids that are morphologically distinct soldiers in the first instar [36] but which nevertheless mature to wingless virginoparae that can produce both winged sexuparae (that migrate from the gall to the poplar bark) and more virginoparae [22,24]. The virginoparous offspring of the second and subsequent galling generations are, once again, soldiers in their first instar. The life cycle described by Foster and Northcott [13] is correct except that we now know that these soldiers produced by the first generation of virginoparae are only facultatively sterile, and can mature to become reproducing virginoparae themselves if sufficient time remains before the leaves of the host plant are shed.

The soldiers of *P. spyrothecae *are among the most aggressive in the genus [15]. They are heavily sclerotised, have enlarged legs and are able to kill predators by puncturing cuticle with their stylets and especially curved claws [24,37]. The presence of soldiers is highly effective in reducing predation on their colony [25].

### Ecological sampling

#### Destructive sampling

Galls were harvested from heights between 1 and 15 m above ground level. These galls were immediately placed in separate sealed vials and taken to the laboratory within 6 h of collection. Each gall was measured to provide for volume calculations and examined for the presence of natural openings or external damage before it was broken open. Aphids were removed from broken galls with a soft brush and scored by instar and morph under a dissecting microscope. An index used for all three species to measure the synchronisation of development of aphids within their galls was created by taking the average proportion of the gall population represented by the predominant morph/instar.

#### Non-destructive monitoring

##### Phenology of the gall

Randomly-selected galls between 1 and 15 m above ground level were marked and numbered with brightly-coloured polyamide tape. At intervals specified below, each gall was externally examined for damage and the presence of natural openings. The dimensions of the gall were taken with a Vernier calliper (accurate to 0.5 mm) to track changes in volume. The apparent condition and quantity of the aphids within the gall was also estimated. Although the precise condition could only be obtained by destructive sampling, it was possible to discern if the aphids were dead or if the gall was empty.

##### Emergence of alates

The galls of a randomly-selected subset of those marked for monitoring were bagged securely with the feet of sheer, white, polyamide stockings. The alates that accumulated in these bags were periodically transferred to sealed polyethylene bags to be counted in the laboratory.

Emergence of alates provides a direct measure of the reproductive success of the galling phase of a clone. In *P. spyrothecae*, each sexupara that emerges from the gall will, if it survives, produce two males and between two and six females, and can thus provide for up to 6 overwintering eggs via which its genes can prevail [38]. In *P. populi *and *P. bursarius*, each fundatrigenia that leaves the gall can found an asexual secondary-host population [32,33].

#### Sampling of *P. populi*

##### Field site

*P. populi *was sampled from a field site (43°34'28" N, 3°49'40" E) in the village of Saint Jean de Védas, France. This small area of parkland contained 30 black and Lombardy poplars.

##### Protocol

*P. populi *was sampled between 1 May and 30 June, 2000. We destructively sampled 20 galls from up to 5 trees three times during this period. 150 galls (75 galls on each of 2 trees) were monitored; and 50 of these (25 galls on each tree) were bagged to gauge emergence of fundatrigeniae.

#### Sampling of *P. bursarius*

##### Field site

The site (52° 11' 2" N, 0° 10' 16" E) near Cambridge from which *P. bursarius *was sampled is a small reserve dominated by a single large Lombardy poplar surrounded by several smaller conspecifics.

##### Protocol

*P. bursarius *was sampled from 28 May-26 July, 2001 at 1–2 week intervals. 30 galls were destructively sampled on seven occasions (a total of 210 galls). 70 galls were marked for monitoring and 50 of these were bagged to measure emergence of fundatrigeniae.

#### Sampling of *P. spyrothecae*

##### Field sites

*P. spyrothecae *was sampled at four field sites in the vicinity of Cambridge, UK. The site 'Coton Footpath' (52° 12' 31" N, 0° 4' 8" E) is a playing field and reserve that contained 10 mature Lombardy poplars (*Populus nigra *var. *italica*). The site 'Cement Works' (52° 11' 49" N, 0° 9' 52" E) is an area of parkland on which more than 50 mature Lombardy poplars grew close together. The 'Leys Playing Field' (52° 11' 22" N, 0° 6' 58" E) is a sports field that contained 16 mature Lombardy poplars. 'Pembroke Sportsground' (52° 11' 25" N, 0° 6' 5" E), also a sports field, contained 20 mature Lombardy poplars.

##### Protocol

*P. spyrothecae *was sampled between 11 May and 22 October, 1999. These dates were used to set the reference frame for interspecific comparisons. Thus, May 11 is set as Day1 and October 22 is set as Day165 for all three species. Destructive sampling and monitoring were conducted simultaneously, at 2–3 week intervals. At each sampling event, 50 galls were destructively sampled: 10 galls (5 from a height of 1 m and 5 from 10 m) were harvested from each of 5 randomly-selected trees. As all the field sites were sampled 9 times, 1800 galls were collected over the entire field season. Fifty marked galls were monitored galls on each of 2 trees at each field site from the beginning of the season until all the aphid colonies under observation had died. Emergence of sexuparae was recorded on a subset of 20 of the 50 monitored galls on each tree. Thus 400 galls were monitored in total, 100 galls at each field site. 40 galls were bagged to measure alate emergence at each site, giving 160 bagged galls across all the sites.

### Construction of phylogeny

#### DNA sequencing

Aphids from eight species of *Pemphigus *(*P. bursarius, P. gairi, P. obesinymphae, P. populi, P. populitransversus, P. protospirae, P. spyrothecae *and *P. trehernei*) were collected and preserved in 95% ethanol. Individual alate aphids were freeze dried and genomic DNA was extracted using standard procedures. An area spanning part of the mitochondrial Cytochrome Oxidase I and II genes was amplified under standard PCR conditions using primers mt2793+ (5'ATACCTCGACGTTATTATTCAGA) and mt3660- (5'CCACAAATTTCTGAACATTGACCA). These, and the following internal primers were used in subsequent re-amplification and sequencing: mt2993+ (5'CATTCATATTCAGAATTACC), mt3175+ (5'CATGAC/TCATACAATTTTTATTAT) and mt3339- (5'GGA/GGATTTAATTTCATCTATT). The internal primers were developed by Stern [39] for use in sequencing *P. microsetosus *and species from the related aphid family Hormaphididae. Primer names indicate position relative to the *Drosophila yakuba *mitochondrial sequence [40].

PCR fragments were visualised by running out on a 2% agarose gel, then purified by cutting the bands from the gel and extracting the DNA by centrifugation followed by a phenol/chloroform extraction. A fraction of each of the resulting samples was re-amplified with internal primers and ethanol precipitated to obtain DNA for sequencing. Automated DNA sequencing was carried out using the ABI Prism dye terminator cycle sequencing kit with AmpliTaq DNA polymerase FS (Perkin-Elmer) and the ABI 310 genetic analyser (Perkin-Elmer). Results were analyzed and sequences aligned by hand using Seqed v. 1.03 (Applied Biosystems), with further sequence editing in preparation for phylogenetic analysis done using MacClade v. 3.05 [41]. The sequence of *P. microsetosus*, which had previously been used in Stern's phylogenetic analysis [39] was obtained from the author and also included in the analysis.

766–845 b.p. were sequenced for each species. Length variation was included in subsequent analyses, with deletions considered as a "fifth base". Length variation consisted of a single base deletion at base 223 in *P. bursarius*, *P. obesinymphae*, *P. microsetosus *and *Eriosoma ulmi*, within the tRNA region. Where bases could not be resolved accurately, the standard IUPAC codes for base uncertainties were used. All ten sequences were submitted to the EMBL/GenBank/DDBJ nucleotide databases [EMBL:AM748711–AM748720].

#### Phylogenetic analysis

Four different techniques were used to estimate the phylogeny: (1) maximum parsimony was implemented with the heuristic algorithm of the computer program PAUP v. 3.1.1 [42] using 10 random addition sequences and all other options as default; (2) neighbour joining was performed using the NEIGHBOR program of the PHYLIP v. 3.53 software suite [43] and maximum likelihood distances for the neighbour joining algorithm were calculated with the DNADIST program of PHYLIP with a transition/transversion ratio of 0.72 and four categories of substitution rate for the three codon positions (0.40, 0.16, and 0.56) and the tRNA region (0.07); (3) the maximum likelihood phylogeny was estimated with the program fastDNAml v. 1.1 [44] using a global search starting from the maximum parsimony tree and the transition/transversion ratio and categories of substitution rate used in the neighbour joining analysis; and (4) Bayesian inference of phylogeny was performed using MrBayes v. 3.1.2 [45]. The transition/transversion ratio was fixed at 0.7 but otherwise default options were accepted for priors (flat priors for the rate matrix and branch lengths, uniform gamma shape parameter, equal site-specific rates) and Markov-Chain Monte Carlo parameters (four concurrent Markov chains and two runs which, after full convergence, were sampled every 100th generation).

All trees were rooted by comparison with the predefined outgroup *Eriosoma ulmi *from the pemphigine tribe Eriosomatini.

## Authors' contributions

NP designed, conducted and analysed the ecological research, contributed to the phylogenetic analysis, and prepared the manuscript. JAW designed and conducted the molecular research, and contributed to the phylogenetic analysis and manuscript. WAF assisted in planning the research and contributed to the manuscript. All authors approved the manuscript.
